# Sensory-guided and statistical characterization of key odorants driving floral differentiation in three Anhui green teas

**DOI:** 10.1016/j.fochx.2026.103936

**Published:** 2026-04-30

**Authors:** Jieyao Yu, Tianyuan Yang, Qian Chen, Gang Wang, Dongzhu Huang, Małgorzata Starowicz, Xiaochun Wan, Xiaoting Zhai

**Affiliations:** a State Key Laboratory of Tea Plant Germplasm Innovation and Resource Utilization, Anhui Agricultural University, Hefei 230036, China; b International Joint Laboratory on Tea Chemistry and Health Effects of Ministry of Education, Anhui Agricultural University, Hefei 230036, China; c Team of Chemistry and Biodynamics of Food, Institute of Animal Reproduction and Food Research, Polish Academy of Sciences, Trylińskiego Street 18, 10-683 Olsztyn, Poland

**Keywords:** Green tea, Floral aroma, Multivariate analysis, Differentiation analysis

## Abstract

The “floral” aroma is highly valued in green tea quality and has attracted growing interest in green tea flavor research recently. However, comparative studies among them remain limited. In this study, three representative floral green teas from Anhui—*Taiping Houkui* (TPHK), *Shucheng Xiaolanhua* (SCXLH), and *Lu’an Guapian* (LAGP)—were analyzed using the sensomics approaches. Multi-criteria evaluation (OAV, PLS-DA, correlation) screened out five key odorants—linalool, geraniol, methyl epijasmonate, *δ*-valerolactone, and jasmone—as major contributors to floral differentiation. Jasmone consistently presented across all analytical dimensions, indicating its key role in both floral intensity and style. Correlation analysis based on lexicon frequency-weighted sensory attributes further revealed that floral style was shaped by not only floral-related odorants but also enhancing (e.g. lactones) and antagonistic (e.g. aldehydes, sulfur compounds) odorants. These findings provide molecular insights into the floral characteristics of Anhui green teas and offer a chemical basis for future efforts to optimize desirable floral profiles.

## Highlights

• Floral aroma of 3 Anhui green teas were characterized by sensomic-approaches.
• A frequency-based weighting was applied to improve sensory scoring reliability.
• 5 odorants were identified as key contributors to the differentiation in floral.
• Jasmone was verified as the most important odorant by multivariate analysis.
• Sweet-related notes could modulate the floral style of green tea indirectly.

## 1 Introduction

Green tea, a non-fermented product of *Camellia sinensis*, is globally favored for its refreshing taste and health benefits. According to ISO 20715:2023, green tea is characterized by the absence of enzymatic oxidation during processing. The typical manufacturing process includes spreading, fixation, rolling, and drying, with the high-temperature fixation rapidly inactivating endogenous enzymes (Yin et al., 2022). This preserves antioxidant phytochemicals such as catechins and other polyphenols, (Luo, Luo, Zhao, Wang, & Luo, 2024; Yu, Zhang, Wang, Zhai, & Wan, 2023), but also limits some transformation of aroma precursors. As a result, green tea generally exhibits lower aroma complexity and diversity in green tea relative to black or oolong teas (Feng et al., 2019; X. T. Zhai, Zhang, Granvogl, Ho, & Wan, 2022), making differentiation among green teas more dependent on subtle variations in key odorants.

Anhui province, one of China's most renowned tea-producing regions, provides an important case for examining the diversification of floral aroma profiles in non-fermented teas. Its diverse ecological environments—from high-altitude Huangshan area to the humid Dabieshan region—are conducive to the accumulation of distinct aroma precursors in tea leaves. In addition to these ecological factors, region-specific processing steps may further influence the transformation of these precursors into odor-active compounds. For example, Anhui green teas are often produced with multi-stage drying processes, which may promote thermal reactions and the release or formation of volatile aroma compounds during drying (Yu et al., 2024; Zhang et al., 2026). The combined influence of environmental conditions and processing characteristics is therefore considered to contribute to the distinctive aroma profiles observed among Anhui green teas. For instance, *Taiping Houkui* is known for its unique orchid-like aroma note (Feng, Li, Li, Wan, & Yang, 2020), the premium grade of *Lu’an Guapian* shows a much stronger floral odor note compared to the common grade (Yu et al., 2023), and *Shucheng Xiaolanhua* also derives its name from its elegant orchid-like scent. Despite the considerable overlap in odorant compositions among green teas, many varieties still exhibit distinctive floral aroma characteristics. These sensory differences may arise from the complex combined effects of cultivar, ecological conditions, and processing. While these factors influence aroma formation, sensory perception is ultimately determined by odor-active compounds. However, the chemical basis underlying these differences—particularly which odorants or quantitative variations contribute to floral differentiation—remains insufficiently understood.

Advances in analytical techniques have significantly improved the undersranding of tea aroma in recent years. The sensomics approach, which integrates instrumental analysis with human sensory evaluation, has proven particularly effective in decoding the key aroma-active compounds in different teas (H. Wang et al., 2024; J. Wang et al., 2022; Yu et al., 2023). Although hundreds of volatile compounds have been reported in green tea, only a small fraction significantly contribute to overall aroma perception—typically those whose concentrations exceed their respective odor thresholds (X. T. Zhai et al., 2022).

Previous studies have identified 122 key odorants across 23 green teas, and surprisingly, a high degree of compositional overlap has been observed (Yin et al., 2022). Common contributors such as 2-methylbutanal, dimethyl sulfide, (*E,E*)-2,4-heptadienal, linalool, geraniol, and (*E*)-*β*-ionone have repeatedly reported as key odorants in representative green teas such as *Longjing*, *Jingshan Cha*, *Laoshan*, and *Lu’an Guapian* (Flaig, Qi, Wei, Yang and Schieberle, 2020a, Flaig, Qi, Wei, Yang and Schieberle, 2020b; Yu et al., 2023; Zhu, Niu, & Xiao, 2021). This compositional similarity makes it difficult to distinguish green tea subtypes solely based on constitute profiles, particularly in terms of their floral characteristics, which often depend on subtle quantitative differences or interactions among odorants.

These floral attributes not only influence consumer preference but also contribute to their higher market value. However, despite extensive investigation into green tea aroma, the underlying chemical basis for these distinctions remain poorly understood. Most previous studies have focused on individual tea types (Kun et al., 2025; Y. Wang et al., 2024; Xie et al., 2023), while the diversity in sample preparation and extraction methods further complicate cross-study comparsion.

Thus, this study aims to identify and compare the key odorants responsible for floral aroma of three representative Anhui green teas: *Taiping Houkui* (TPHK), *Shucheng Xiaolanhua* (SCXLH), and *Lu’an Guapian* (LAGP). A combination of solvent-assisted flavor evaporation (SAFE) and headspace-solid-phase microextraction (HS-SPME) was used for aroma extraction, followed by sensory evaluation, GC–MS/O analysis, aroma extract dilution analysis (AEDA), and multivariate statistical analyses including odor activity value (OAV) and partial least squares discriminant analysis (PLS-DA), to clarify the characteristic odorants that differentiate floral profiles among the selected floral green teas. This work seeks to provide an understanding of the representative floral-type green teas from Anhui in order to elucidate the molecular basis of their floral differentiation in aroma.

## 2 Materials and methods

### 2.1 Reagents and chemicals

The chemicals were all commercially obtained and were chromatographically pure (>95%) unless otherwise stated. The dichloromethane (DCM) were obtained from TEDIA, Shanghai, China, and rectified before use. The diethyl ether (DEE), NaCl, and Na_2_SO_4_ were obtained from Sinopharm Chemical Reagent Co., Ltd., Shanghai, China. The *n*-alkane mixtures (C_5_-C_35_) were purchased from Aladdin Chem., Shanghai, China. The silica gel G_60_ (230–400 mm, 99.5%) was obtained from Alfa Aesar, Shanghai, China.

The reference aroma compounds for identification and quantitation experiments were commercially obtained and were at least 95% pure for GC: methional, (*E,Z*)-2,6-nonadienal, 2-methylpropanal, 2/3-methylbutanal, 4-hydroxy-4-methylpentan-2-one, (*Z*)-3-hexen-3-ol, 2-phenylethanol, 2-phenoxyethanol, 6-methyl-5-hepten-2-one, (*E*)-*β*-ionone, jasmone, 4-(4-hydroxyphenyl)-2-butanone, butanoic acid, 3-methyl butanoic acid, dodecanoic acid, phenylacetic acid, *γ*-butyrolactone, *γ*-hexalactone, *δ*-valerolactone, *γ*-octalactone, *δ*-octalactone, *γ*-decalactone, methyl anthranilate, jasmine lactone, methyl jasmonate (MeJA), dihydroactinidiolide, methyl epijasmonate (epi-MeJA), 3-hydroxy-2-methyl-4H-pyran-4-one, 4-methylphenol, 4-hydroxy-2,5-dimethyl-3(2*H*)-furanone, 3-ethyl-4-methyl-pyrrole-2,5-dione, 7-methoxycoumarin, dimethyl sulfide were obtained from Macklin Biochem, Shanghai, China. Hexanal, (*Z*)-4-heptenal, benzaldehyde, methanethiol, benzyl alcohol, linalool, geraniol, hexanoic acid, nonanoic acid, methyl salicylate, and phenol were purchased from Aladdin Chem,. Shanghai, Cina. Indole and coumarin were obtained fom Dr. Ehrenstorfer GmbH, Augsburg, Germany, while 2/3-methylbutanal were obtained from TCI, Shanghai, China. (*E,E*)-2,4-Heptadienal, vanillin, and *δ*-decalactone were purchased from Ark Pharm (Shanghai, China), Shanghai Yuanye Co., Ltd. (Shanghai, China), and TMstandard (Changzhou, China) respectively.

### 2.2 Samples and tea infusion preparation

The tea samples were collected from a local market and manufacturers in Anhui Province, China. Detailed information of the samples was provided in Table S1, and the typical processing procedures were illustrated in Fig. S1. For each type, multiple commercial samples were initially screened, and one representative sample was selected for detailed analysis based on its sensory characteristics and overall quality. The samples were stored at −20 °C before analysis.

The samples were brewed according to a previous method (Yu et al., 2023) with some modifications. Tea leaves (6 g) were infused with 100 mL pure water (95 °C) in a conical flask first, then filtered after a three-minute brewing. Next, 50 mL pure water (room temperature) was added to flush the leaves and combined with the previous part, then the combination was put into an ice bath to cool down the temperature.

### 2.3 Isolation of volatile fractions from tea infusion

#### 2.3.1 Solvent assistant flavor extraction (SAFE)

After cooling, the infusions were extracted with 300 mL DCM, and the organic phase was separated via the separatory funnel. The extractions were concentrated to 50–100 mL for SAFE (Engel, Bahr, & Schieberle, 1999). The distillate was dried with anhydrous sodium sulfate before being concentrated (Vigreux-column, 60 cm × 1 cm i.d.) for de-caffeine. The caffeine was removed by flash chromatography described in previous literature (M. Flaig et al., 2020a). Finally, the Vigreux column (60 cm × 1 cm i.d.) and micro-distillation apparatus were used to concentrate the eluate to 200 μL for the follow-up analysis.

#### 2.3.2 Headsapce solid-phase microextraction (HS-SPME)

The volatiles with higher volatility in tea infusion tend to partially volatilize during the liquid-liquid extraction and were easily covered in the solvent peak during analysis. Thus, HS-SPME was used for the capture and enrichment of this fraction to analyze the tea aroma comprehensively while avoiding being covered by the solvent peak. The method was conducted according to Feng et al. (2020) with some slight modifications. The infusions were brewed as described above, and swiftly cool down in an ice bath. Then, a 10 mL infusion was moved into a sealed headspace bottle (20 mL) with 2 g NaCl and incubated in a water bath (30 °C) for 15 min. Next, the HS-SPME stable flex fiber (polydimethylsiloxane/divinylbenzene, PDMS/DVB, 65 μm, Oakville, Canada) fitted on a manual SPME holder was inserted into the vial for a 30 min headspace absorption.

### 2.4 Gas chromatography-olfactometry (GC-O) analysis

#### 2.4.1 Instrument conditions

The analysis was performed by a gas chromatography (7890B, Agilent, CA, USA) equipped with a flame ionization detector (FID) and connected with an olfactory detection port (Gerstel, ODP3, Mülheim an der Ruhr, Germany). The carrier gas of the system was helium (99.999% pure). The samples were injected into the injector (250 °C) with splitless mode. The analytical conditions for DB-FFAP (30 m × 0.25 mm × 0.25 μm, Agilent, CA, USA) were as follows: the oven temperature was held at 40 °C for 5 min, then heated at 6 °C/min to 150 °C; then heated at 4 °C/min to 230 °C and maintained for 17 min. The analytical conditions for DB-5MS (30 m × 0.25 mm × 0.25 μm, Agilent, CA, USA) were as follows: the oven temperature was held at 40 °C for 5 min, then heated at 8 °C/min to 100 °C; heated at 4 °C/min to 200 °C; heated at 10 °C/min to 280 °C and maintained for 5 min. The carrier gas was equally split to the FID (250 °C) and ODP (200 °C) via an Agilent deanswitch.

#### 2.4.2 Aroma extract dilution analysis (AEDA)

The aroma-active compounds were determined through three trained assessors by sniffing the original concentrated aroma distillate at the ODP. Then, the DCM was used to dilute the distillate 1:1 by volume stepwise. The AEDA was analyzed with a DB-FFAP capillary column. The diluted samples were injected into GC-O from high to low concentrations, and the assessors were required to record the last dilution step when no odorants could be detected at the sniff port. The final dilution fold on average of three assessors was the corresponding FD (flavor dilution) factor of the aroma-active compounds.

### 2.5 Gas chromatography-mass spectrometry (GC–MS) analysis

The identification of volatiles was conducted by gas chromatograph-mass spectrometry (7890B—5977B, Agilent, CA, USA). The samples were injected or inserted into a DB-FFAP or DB-5MS column in a splitless mode. The chromatographic conditions were the same as mentioned above. The mass spectrometer conditions were as follows: electron impact mode (MS-EI); ion source temperature, 230 °C; ionization energy, 70 eV; mass scan range, 30–350 *m/z*.

### 2.6 Quantitation of volatiles

A primary experiment was conducted to determine the proper concentration of the internal standard. Then, 10 μL ethyl decanoate (10–20 μg in DCM) was added into the infusions before extraction and isolation as the internal standard. The corresponding standard curves were built with reference aroma compounds and ethyl decanoate at different concentration ratios (5:1, 3:1, 1:1, 1:3, and 1:5). For the volatiles extracted by HS-SPME, the quantitation was conducted in a consistent extraction. Ethyl decanoate was added to the infusions and adsorbed along with the infusions by the fiber for 30 min after a fifteen-minute incubation both in a 30 °C water bath. Then the fiber was inserted into GC–MS for analysis under the same conditions above mentioned. The information about standard curves is shown in Table S2.

### 2.7 Sensory evaluation and weighting

#### 2.7.1 Sensory evaluation

Sensory analysis was conducted in a dedicated sensory evaluation room. The brewing conditions for sensory evaluation were in accordance with the infusions prepared for chemical analysis, i.e. 3 g tea with 75 mL 95 °C pure water, brewing for 3 min. A group of 13 well-trained panelists from the School of Tea and Food Science and Technology (Anhui Agricultural University, Anhui, China) participated in sensory evaluation. The infusions were first assessed by Chinese traditional sensory analysis as described in GB/T 23776–2018. at room temperature. The panelists were first required to freely generate descriptors for each sample, and attributes were subsequently selected based on their frequency of occurrence. After the determination of the descriptors for the aroma of the samples, corresponding reference aroma compounds were provided as the standard in concentration at 100 folds of their thresholds. The compounds used included hexanal (green, grassy), MeJA (orchid-like), geraniol (floral), 2-acetylpyrazine (roasty/popcorn-like), dimethyl sulfide (cooked vegetable-like), *δ*-decalactone (sweet), (*E,Z*)-2,6-nonadienal (fresh), linalool (tender), (*E,E*)-2,4-decadienal (fatty), and 3-methylnonane-2,4-dione (hay-like). All reference compounds were initially dissolved in ethanol and subsequently diluted in pure water. Then, the samples were served with random code to panelists in glass vessels to score the intensity of each attribute. The score range was 0 to 3, while 0 means “barely sensed”, 1 means “could be sensed but hard to recognize”, 2 means “clearly sensed and recognized”, and 3 means “strongly sensed and recognized”. The final aroma profiles were established based on the average of all panelists.

Ethical approval for this sensory evaluation study was not required according to national regulations, and no formal human ethics committee approval was necessary. The study was conducted in compliance with the Code of Ethics of the World Medical Association (Declaration of Helsinki) for experiments involving human participants.

All participants were informed about the purpose and potential risks of the study prior to participation, and verbal consent was obtained. Participation was voluntary, and participants had the right to withdraw at any time. No personal data were disclosed without consent, and all procedures were designed to ensure the protection of participants' rights and privacy.

#### 2.7.2 Frequency-based weighting

To reflect the relative importance of different aroma descriptors, sensory scores were adjusted using a frequency-based weighting approach for subsequence correlation analysis. Descriptor frequency was selected as the weighting basis as frequently cited descriptors are generally regarded as more important and representative in lexicon development (Drake & Civille, 2003; Suwonsichon, 2019), which providing a rational criterion for emphasizing perceptually relevant attributes relevance. The weighting scheme was further adapted from principles of the entropy weight method (EWM) (Fadlallah, Chen, Keil, & Príncipe, 2013; Gao et al., 2021) and the term frequency-inverse document frequency (TF-IDF) algorithm widely used in numerical text analysis (Aizawa, 2003; Salton & Buckley, 1988; Wu, Luk, Wong, & Kwok, 2008), both of which apply logarithmic scaling to balance the influence of high- and low-frequency terms.

The descriptors were first classified into three groups: “floral”, “de-floral”, and “neutral” (Table S3).

For each attribute *j*, the sensory score of sample *i* was normalized using *Z*-score standardization (Eq. 1):

(1) $$Z_{i,j}=\frac{X_{i,j}-\mu_{j}}{\sigma_{j}}$$

*X*_i,j_: The raw score of sample *i* on *j* attribute.

μ_j_, σ_j_: The mean and standard deviation of attribute *j* across all samples.

The weight of each attribute *j* in sample *i* (W_*i,j*_) was then calculated according to its citation frequncy (Eq. 2):

(2) $$W_{i,j}=\frac{\ln\left(f_{i,j}+1\right)}{\sum_{k=1}^{m}\ln\left(f_{i,k}+1\right)}$$

*f*_*i,j*_: The the number of panelists who cited attribute *j* in *i* sample.

*m*: The total number of attributes in *i* sample.

Finally, the weighted group scores for each sample (S_*i,g*_) were obtained by summing the weighted scores of all descriptors within each group (Eq. 3):

(3) $$S_{i,g}=\sum_{j\in g}Z_{i,j}*W_{i,j}$$

### 2.8 Statistical analysis

All experiments and sample analyses in this study were presented as mean (at least three replicates) ± standard deviation (SD), the latter were all below 20%. The significance analysis was conducted by one-way analysis of variance (ANOVA) using IBM SPSS Statistic software (Version 19.0, SPSS Inc., Chicago, IL, USA). Spearman correlation analysis was conducted to evaluate the relationships between sensory attributes and odor-active compounds (OAV > 1). Prior to analysis, the data were standardized using *Z*-score normalization. Correlation coefficients and their significance levels were calculated using Origin 2023 (OriginLab Corporation, Northampton, MA, USA) statistical significance was set at *p* < 0.05, and no correction for multiple comparisons was applied, as the analysis was considered exploratory.

The circle packing plot was conducted by Chiplot (https://www.chiplot.online/). The heatmap figures were charted by Origin (OriginLab Co., Massachusetts, USA). The partial least squares-discriminant analysis (PLS-DA) was conducted by SIMCA with omics (version 14.1, Umetrics, Sweden). The network relationship plot was performed by Gephi (Version 0.10.0, https://gephi.org/).

## 3 Results

### 3.1 Sensory profiles of three different green teas

**Fig. 1:**
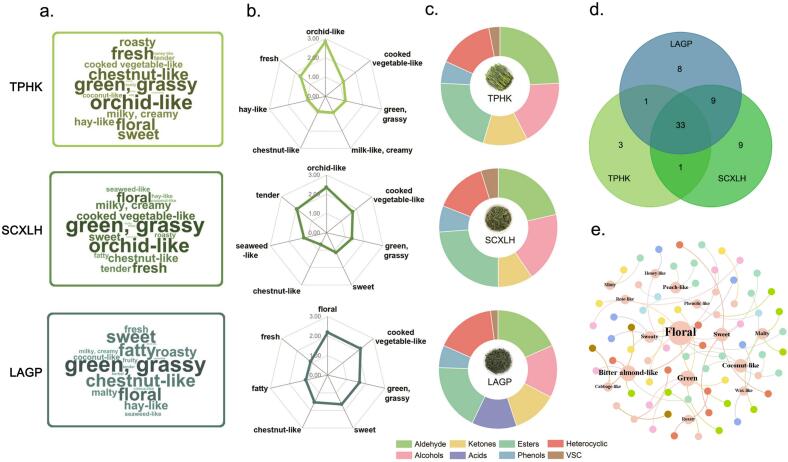
Sensory evaluation (a-b), and overview of volatile compounds (c-e) of three floral green teas. a. Word clouds of *Taiping Houkui* (TPHK, line 1), *Shucheng Xiaolanhua* (SCXLH, line 2), and *Lu’an Guapian* (LAGP, line 3), constructed from descriptor citation frequencies. b. Aroma profiles of TPHK (line 1), SCXLH (line 2), and LAGP (line 3), the scored attributes for different sample were selected based on their citation frequencies respectively. c. Classification of volatile compounds identified by GC-MS/O. d. Venn diagram showing the distribution and overlap of volatile compounds among the teas. e. Network relation map of odorants with their associated odor qualities across the samples. (For interpretation of the references to colour in this figure legend, the reader is referred to the web version of this article.)

To evaluate the differences across the three samples with floral aromas precisely, both traditional sensory assessment (Table S4) and aroma profile analyses were conducted to evaluate the samples. As shown in Fig. 1a, although all three samples have “floral” as their descriptor, the panelists identified differences in the floral attributes when prompted for more detailed descriptions. The floral note in TPHK was identified as “intense orchid-like”, LAGP was described as “delicate floral”, and SCXLH as “bright orchid-like”. These distinctions suggested that, despite a shared floral base, the samples exhibit divergent aroma profiles likely attributed to their varietal characteristics and processing techniques.

For each sample, the top seven descriptors were selected based on high-frequency occurrences (Fig. 1b) to provide a clearer depiction of their respective aroma profiles, further illustrated by radar plots. TPHK (Fig. 1b, line 1) demonstrated the highest intensity in “orchid-like” aroma, reaching the maximum score among all descriptors, followed by “fresh” and “green, grassy” notes. Other aroma attributes such as “chestnut-like”, “milk-like, creamy”, and “hay-like” were relatively low, suggesting that the sample is primarily dominated by a floral and fresh scent profile with minimal influence from other notes. In contrast, SCXLH (Fig. 1b, line 2) showed a more balanced sensory profile. While “orchid-like” remained one of the major descriptors, it also showed moderate intensities of “fresh”, “tender”, and “green, grassy” notes. Additionally, faint notes of “tender chestnut-like” and “seaweed-like” contributed to a more layered and nuanced aromatic structure. The distinct presence of the “tender” note sets this sample apart and may reflect differences in leaf maturity or more delicate processing conditions. LAGP (Fig. 1b, line 3), however, presented a markedly different profile, dominated by “chestnut-like” and “floral” aromas. Unlike TPHK and SCXLH, the “orchid-like” descriptor did not meet the frequency threshold for inclusion. Instead, stronger contributions from “cooked vegetable-like” and “fatty” notes were observed. This profile is consistent with processing stages involving repeated baking (Zhang et al., 2023), which are known to enhance chestnut-like characteristics while diminishing green or floral notes (Yu et al., 2024; Zhang et al., 2024).

The radar plots underscore distinct aroma profiles among the three teas. TPHK exhibited a fresh-floral character dominated by orchid-like notes, SCXLH displayed a delicate, multi-layered floral profile with subtle sweet note, and LAGP was characterized by a roasted-floral aroma with a faint fatty undertones.

### 3.2 Identification of aroma-active compounds

The distillates of TPHK, SCXLH, and LAGP were all evaluated using a fragrance blotter and verified that their overall aroma profiles corresponded with those of the respective tea infusions. Based on the screening results obtained from GC–MS/O of the concentrated aroma distillate with the results of AEDA, a total of 64 aroma-active compounds were detected at the sniffing port with FD factors greater than 16 (Table 1). 51 and 52 aroma-active compounds were detected in SCXLH and LAGP, respectively, while TPHK contained the fewest, with 38 aroma-active compounds. Compound **14** (floral), **26** (rose-like, citrus-like), **30** (floral, violet-like), **53** (mothball-like), and **57** (vanilla-like, sweet) had the highest FD factors (>256) in both three samples, while **52** (floral, orchid-like; 512) only present high FD factor in TPHK. Three odorants, **28** (floral, honey-like), **38** (horse stable-like), and **46** (coconut-like) exhibited high FD values exclusively in the SCXLH, suggesting a stronger association with its subtle floral attribute. Compounds **5** (green, nutty; 512) and **51** (coumarin-like, fruity; 1024) were only perceived in LAGP with high FD factors, these two distinct odorants may contribute to the differentiation of the aroma in LAGP.

**Table 1:** Aroma compounds identified in three green teas and their FD factors obtained by AEDA.

No.^a^	Compounds	Identification^b^	Odor quality^c^	RI^d^	FD^e^		
					TPHK	SCXLH	LAGP
*Aldehydes*							
1	hexanal	MS/RI/O/STD	green, grassy	1068	64	64	32
3	(*Z*)-4-heptenal	RI/O/STD	fish-like, train oil-like	1240	32	32	256
11	methional	RI/O/STD	cooked potato-like	1448	64	32	64
12	(*E,E*)-2,4-heptadienal	MS/RI/O/STD	fatty, floral	1488	n.d.^f^	<16	64
13	benzaldehyde	MS/RI/O/STD	bitter almond-like	1515	64	256	128
16	(*E,Z*)-2,6-nonadienal	MS/RI/O/STD	cucumber-like	1578	n.d.	64	32
57	vanillin	MS/RI/O/STD	vanilla-like, sweet	2557	512	512	2048
HS-3	2-methylpropanal	MS/RI/O/STD	malty	817	1	1	n.d.
HS-4	3-methylbutanal	MS/RI/O/STD	malty	923	1	4	4
HS-5	2-methylbutanal	MS/RI/O/STD	malty	928	1	8	16
*Alcohols*							
2	3-penten-2-ol	MS/RI/O	green	1161	n.d.	32	32
8	(*Z*)-3-hexen-1-ol	MS/RI/O/STD	green, apple-like	1372	256	256	128
14	linalool	MS/RI/O/STD	floral	1533	512	1024	512
22	trans-linalool oxide (IV)	MS/RI/O	floral, wintergreen	1750	n.d.	32	64
26	geraniol	MS/RI/O/STD	rose-like, citrus-like	1834	1024	1024	512
27	benzyl alcohol	MS/RI/O/STD	bitter almond-like	1866	16	32	16
28	2-phenylethanol	MS/RI/O/STD	floral, honey-like	1900	64	512	256
39	2-phenoxyethanol	MS/RI/O/STD	rose-like	2133	16	32	32
*Ketones*							
4	1-octen-3-one	MS/RI/O	mushroom-like	1299	16	16	16
5	6-methyl-5-hepten-2-one	MS/RI/O/STD	green, nutty	1328	n.d.	n.d.	512
10	2-hydroxy-3-hexanone	MS/RI/O	green, hay-like	1421	n.d.	n.d.	32
30	(*E*)-*β*-ionone	MS/RI/O/STD	floral, violet-like	1923	1024	512	512
31	jasmone	MS/RI/O/STD	coconut-like, floral	1933	128	512	64
59	4-(4-hydroxyphenyl)-2-butanone	MS/RI/O/STD	raspberry-like	2981	128	2048	1024
*Acids*							
18	butanoic acid	MS/RI/O/STD	sweaty	1616	n.d.	n.d.	64
20	3-methyl butanoic acid	MS/RI/O/STD	sweaty	1656	n.d.	n.d.	64
25	hexanoic acid	MS/RI/O/STD	sweaty	1831	n.d.	<16	64
42	nonanoic acid	MS/RI/O/STD	moldy, fatty	2155	n.d.	<16	16
55	dodecanoic acid	MS/RI/O/STD	wax-like	2472	n.d.	n.d.	16
56	phenylacetic acid	MS/RI/O/STD	beeswax-like	2550	n.d.	<16	256
*Esters*							
19	*γ*-butyrolactone	MS/RI/O/STD	cheese-like, sweet	1624	n.d.	16	16
21	*γ*-hexalactone	MS/RI/O/STD	coconut-like, fruity	1695	256	2048	1024
23	methyl salicylate	MS/RI/O/STD	minty, terpene-like	1767	16	<16	<16
24	*δ*-valerolactone	MS/RI/O/STD	coconut-like, sweet	1801	n.d.	128	n.d.
29	*γ*-octalactone	MS/RI/O/STD	coconut-like	1912	n.d.	<16	32
32	*δ*-octalactone	MS/RI/O/STD	coconut-like	1961	n.d.	32	n.d.
37	dimethyl salicylate	MS/RI/O	herbaceous, floral	2062	n.d.	32	n.d.
40	*γ*-decalactone	MS/RI/O/STD	peach-like, coconut-like	2134	n.d.	256	n.d.
44	*δ*-decalactone	MS/RI/O/STD	coconut-like	2183	128	1024	1024
45	methyl anthranilate	MS/RI/O/STD	fruity	2229	64	<16	32
46	jasmine lactone	MS/RI/O/STD	coconut-like, floral	2246	64	1024	64
49	methyl jasmonate	MS/RI/O/STD	floral, orchid-like	2319	512	32	32
51	dihydroactinidiolide	MS/RI/O/STD	coumarin-like, fruity	2339	n.d.	n.d.	1024
52	methyl epijasmonate	MS/RI/O/STD	floral, orchid-like	2375	2048	2048	256
*Phenols*							
33	3-hydroxy-2-methyl-4*H*-pyran-4-one	MS/RI/O/STD	caramel-like	1964	<16	n.d.	32
34	phenol	MS/RI/O/STD	ink-like	1997	<16	32	64
38	4-methylphenol	MS/RI/O/STD	horse stable-like	2072	64	512	256
50	trans-isoeugenol	MS/RI/O	clove-like	2338	16	256	<16
*Heterocyclic compounds*							
6	2-acetyl-1-pyrroline	MS/RI/O	popcorn-like, roasty	1329	128	n.d.	n.d.
7	4-hydroxy-4-methylpentan-2-one	MS/RI/O/STD	minty	1351	n.d.	128	n.d.
15	2-propionylfuran	MS/RI/O	green, nutty	1563	n.d.	n.d.	16
35	4-hydroxy-2,5-dimethyl-3(2*H*)-furanone	MS/RI/O/STD	caramel-like	2020	256	1024	2048
36	nerol oxide	MS/RI/O	floral	2030	n.d.	n.d.	128
47	3-ethyl-4-methyl-pyrrole-2,5-dione	MS/RI/O/STD	sweet, caramel-like	2257	<16	64	32
53	indole	MS/RI/O/STD	mothball-like	2429	512	2048	2048
54	coumarin	MS/RI/O/STD	almond paste-like	2446	128	1024	1024
58	7-methoxycoumarin	MS/RI/O/STD	bitter almond-like	2939	32	64	256
*Sulfur compounds*							
HS-1	methanethiol	RI/O/STD	cabbage-like	<800	n.d.	1	n.d.
HS-2	dimethyl sulfide	MS/RI/O/STD	corn-like	<800	16	32	32
*Unkown*							
9	unkown 1	O	fatty, rice-like	1409	n.d.	64	n.d.
17	unkown 2	O	floral	1605	32	n.d.	n.d.
41	unkown 3	O	leather-like	2141	n.d.	32	n.d.
43	unkown 4	O	herbaceous	2160	64	n.d.	n.d.
48	unkown 5	O	soapy, citrus-like	2288	n.d.	32	n.d.

a Numbers of volatile compounds were sorted based on the RI on the DB-FFAP column.

b Identification method: MS, mass spectra; RI, retention index; O, olfactometry; STD, authentic reference compounds.

c Odor quality were determined at the sniffing port.

d The RI on DB-FFAP column was calculated by a mixture of *n*-alkane series (C_5_-C_35_).

e FD factors of odorants in three samples determined by AEDA on the DB-FFAP column.

f Not detected at the sniffing port.

To identify the corresponding compounds for these odor impressions, the RIs were calculated and compared with reported references along with the mass spectrogram (NIST 20.0). Following a preliminary determination through RIs, the recorded odor impressions were compared to the standard compounds, especially the odor quality and retention time on the same instrument. Except for compounds **9**, **17**, **41**, **43**, and **48**, fifty-nine odorants were identified by at least compliance with three qualitative standards. A network map visualizing the connections between the 59 identified odorants and their associated odor qualities was created by Gephi 0.10.0 software (Fig. 1e). This network representation effectively captures the complexity and interrelationships among different odor qualities in the sample, with distinct clusters revealing potential groupings of compounds that share similar odor attributes. Nodes in the network represent odor qualities, with their size corresponding to the number of connected odorants. Larger nodes, such as “floral” and “green”, indicate a higher number of associated odorants. The edges between nodes illustrate the relationships between these odor qualities, with different colors representing distinct categories of related odorants. The “floral” node appears central and highly interconnected, indicating its importance as a primary odor quality in the volatile profile. Other significant odor notes, such as “sweet”, “bitter almond-like”, and “coconut-like”, also display a relatively high number of connections. Additionally, smaller nodes like “minty” and “rose-like” suggest fewer associated compounds.

Nine odorants, including (*E*,*E*)-2,4-heptadienal, linalool, (*E*)-*β*-ionone, jasmone, MeJA, epi-MeJA, (*E*)-isoeugenol, trans-linalool oxide (IV), and nerol oxide contributed to the “floral” odor note. These compounds exhibited relatively high FD factors and further substantiated their key roles in shaping the floral attributes across the three samples. Notably, the “rose-like” node, while representing a more specific olfactory impression, falls under the broader category of floral attributes. Odorants contributing to this note can be regarded as a subclass of floral compounds with more narrowly defined sensory characteristics.

### 3.3 Quantitation of aroma-active compounds

Although AEDA helped narrow down the aroma-active compounds, the matrix in which they are present could influence their release and subsequently affect the overall aroma profile. Therefore, further quantification is necessary to better assess the contribution of these odorants.

A total of 62 compounds were quantified based on extracted ion chromatograms (EIC) using ethyl decanoate as the internal standard and external calibration curves (Table S2). While LAGP contained the largest variety of odorants (41 compounds; 693.51 μg/L), the total concentration of odorants was highest in SCXLH (33 compounds; 918.00 μg/L). In TPHK, 27 odorants were quantified, with a cumulative concentration of 574.54 μg/L. Among these, MeJA exhibited the highest concentrations, with similar levels detected in both TPHK (270.94 μg/L) and SCXLH (270.81 μg/L). This was followed by dimethyl sulfide (37.32 μg/L) and 4-hydroxy-4-methylpentan-2-one (192.90 μg/L). Notably, dimethyl sulfide was also found in high concentrations in LAGP (84.40 μg/L), second only to hexanoic acid (91.34 μg/L). Odorants strongly associated with a “green” odor note were present in significantly higher concentrations in LAGP. For example, hexanal (green, grassy) reached a concentration of 34.01 μg/L, while (*Z*)-3-hexen-1-ol (green, apple-like aroma) was quantified at 27.97 μg/L. Interestingly, indole, which imparts a distinctive mothball-like aroma, was significantly higher in LAGP compared to the other two samples. This compound has also been identified as a characteristic odorant in *Lu’an Guapian* green tea (Yu, Ho, et al., 2023). Benzyl alcohol, typically associated with a honey-like aroma in tea (H. Wang, Bi, et al., 2024), reached 42.56 μg/L in SCXLH, 36.34 μg/L in TPHK, and 27.01 μg/L in LAGP. In TPHK, MeJA was the dominant odorant, accounting for 47.16% of the total quantified odorants. Although SCXLH had a similar concentration of MeJA, it only represented 29.50% of the total odorants. This compound is closely associated with an “orchid-like” aroma, which helps explain why TPHK has the most pronounced orchid-like fragrance among the three floral green tea samples.

**Fig. 2:**
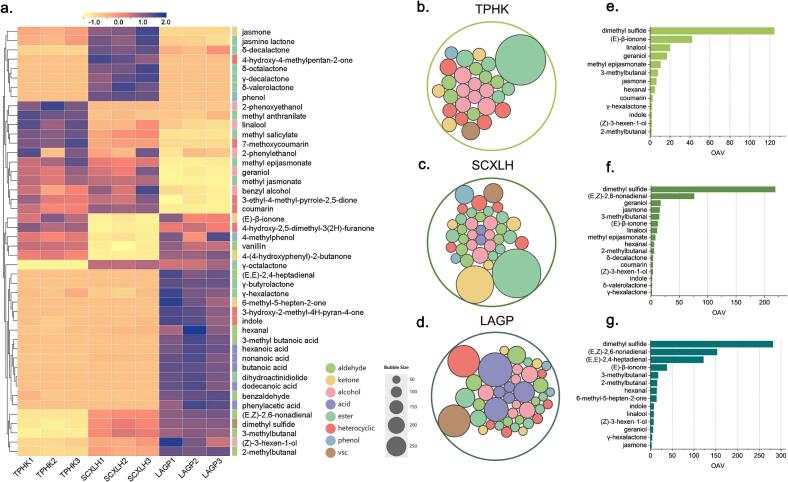
Visualization of quantified odorants in three floral green teas. a. Heatmap of odorant concentrations across samples. b-d. Circle packing plot showing the distributions of quantified odorants in the three floral green teas. e-g. Odor-active compounds (OAV > 1) ranked by their OAVs from highest to lowest. (For interpretation of the references to colour in this figure legend, the reader is referred to the web version of this article.)

Although there were significant differences in both the different odorants in the same sample and the same odorants between samples, some odorants with low detection thresholds (ODT) still made substantial contributions to the overall aroma, even at low concentrations. Thus, odor activity values (OAVs) were calculated to better evaluate the impact of these odorants on samples aroma profiles (Table 2). The full quantitative dataset is summarized in Table 2, while Fig. 2 presents the results visually.

**Table 2:** Concentrations and OAVs of odorants identified in three floral green teas.

No.^A^	Compounds	Concentration (μg/L)^B^			OT (μg/kg)^C^	OAV^D^		
		TPHK	SCXLH	LAGP		TPHK	SCXLH	LAGP
HS-2	dimethyl sulfide	37.32^a^	65.71^b^	84.40^c^	0.3	124.4	219.0	281.3
HS-4	3-methylbutanal	11.51^a^	21.78^b^	26.54^c^	1.5	7.7	14.5	17.7
HS-5	2-methylbutanal	2.17^a^	8.18^b^	22.84^c^	1.5	1.4	5.5	15.2
1	hexanal	11.58^a^	13.12^a^	34.01^b^	2.4	4.8	5.5	14.2
5	6-methyl-5-hepten-2-one	n.d^aD^	n.d^a^	2.22^b^	0.16	-^E^	–	13.9
7	4-hydroxy-4-methylpentan-2-one	n.d^a^	192.90^b^	n.d^a^	100,000	–	<1	–
8	(*Z*)-3-hexen-1-ol	6.46^a^	13.10^a^	27.97^b^	3.9	1.7	3.4	7.2
12	(*E,E*)-2,4-heptadienal	n.d^a^	-^aF^	3.90^b^	0.032	–	–	121.9
13	benzaldehyde	2.99^a^	2.71^a^	11.61^b^	150	<1	<1	<1
14	linalool	11.64^c^	6.28^b^	4.36^a^	0.58	20.1	10.8	7.5
16	(*E,Z*)-2,6-nonadienal	n.d^a^	0.34^b^	0.69^c^	0.0045	–	76.2	153.1
18	butanoic acid	n.d^a^	n.d^a^	9.33^b^	2400	–	–	<1
19	*γ*-butyrolactone	n.d.^a^	0.10^a^	38.37^b^	50	–	<1	<1
20	3-methyl butanoic acid	n.d^a^	n.d^a^	7.36^b^	490	–	–	<1
21	*γ*-hexalactone	3.00^a^	2.67^a^	6.21	1.6	1.9	1.7	3.9
23	methyl salicylate	2.09^c^	1.30^b^	-^a^	24	<1	<1	–
24	*δ*-valerolactone	n.d^a^	16.25^b^	n.d^a^	8.4	–	1.9	–
25	hexanoic acid	n.d^a^	1.12^a^	91.34^b^	4800	–	<1	<1
26	geraniol	18.48^a^	18.55^a^	6.72^b^	1.1	16.8	16.9	6.1
27	benzyl alcohol	36.34^b^	42.56^c^	27.01^a^	620	<1	<1	<1
28	2-phenylethanol	30.18^a^	28.50^a^	25.29^a^	140	<1	<1	<1
29	*γ*-octalactone	n.d^a^	0.26^b^	0.25^b^	6.5	–	<1	<1
30	(*E*)-*β*-ionone	0.88^b^	0.26^a^	0.80^b^	0.021	42.1	12.2	37.9
31	jasmone	11.93^b^	29.42^c^	5.02^a^	1.9	6.3	15.5	2.6
32	*δ*-octalactone	n.d^a^	0.19^b^	n.d^a^	100	–	<1	–
33	3-hydroxy-2-methyl-4H-pyran-4-one	-^a^	n.d^a^	5.54^b^	5000	–	–	<1
34	phenol	-^a^	61.80^b^	2.58^a^	3400	–	<1	<1
34	4-hydroxy-2,5-dimethyl-3(2*H*)-furanone	8.57^c^	1.14^a^	6.83^b^	37	<1	<1	<1
38	4-methylphenol	0.24^ab^	0.17^a^	0.30^b^	3.9	<1	<1	<1
39	2-phenoxyethanol	2.64^b^	0.35^a^	0.20^a^	354	<1	<1	<1
41	*γ*-decalactone	n.d^a^	0.40^b^	n.d^a^	24.8	–	<1	–
43	nonanoic acid	n.d^a^	2.65^a^	55.64^b^	26	–	<1	2.1
44	*δ*-decalactone	0.25^a^	6.73^c^	1.88^b^	1.8	<1	3.7	1.0
45	methyl anthranilate	0.88^b^	0.25^a^	0.24^a^	1500	<1	<1	<1
46	jasmine lactone	10.98^b^	22.92^c^	7.12^a^	120	<1	<1	<1
48	3-ethyl-4-methyl-pyrrole-2,5-dione	2.21^b^	2.35^b^	0.91^a^	180	<1	<1	<1
50	methyl jasmonate	270.94^b^	270.81^b^	27.53^a^	5700	<1	<1	<1
51	dihydroactinidiolide	n.d^a^	n.d^a^	1.72^b^	8856	–	–	<1
52	methyl epijasmonate	31.25^c^	23.35^b^	3.48^a^	3	10.4	7.8	1.2
53	indole	20.19^a^	22.82^a^	83.05^b^	11	1.8	2.1	7.5
54	coumarin	27.49^b^	37.52^c^	2.40^a^	11	2.5	3.4	<1
55	dodecanoic acid	n.d^a^	n.d^a^	44.34^b^	10,000	–	–	<1
56	phenylacetic acid	n.d^a^	-^a^	10.89^b^	68	–	–	<1
57	vanillin	0.75^b^	0.40^a^	0.78^b^	53	<1	<1	<1
58	7-methoxycoumarin	13.59^c^	4.58^b^	1.54^a^	72	<1	<1	<1
59	4-(4-hydroxyphenyl)-2-butanone	0.21^a^	0.04^a^	0.28^c^	1	<1	<1	<1

A The numbers are in accordance with Table 1.

B Concentrations were present as “average concentration ± SD”, including three replicates. Significance analysis was conducted by a *t*-test, values with different lowercase letters in same line represent significant difference (*p* < 0.05).

C Odor thresholds (OTs) were obtained from the Leibniz-LSB@TUM Odorant Database (http://www.leibniz-lsb-de/en/database) and an in-house database.

D The odor activity values (OAVs) were calculated by dividing the average concentration by the OTs in water.

The ranking of odorants with odor activity values (OAVs) greater than 1 differed notably from that based on concentrations. In descending order, the number of odorants with OAV > 1 was highest in LAGP (17), followed by SCXLH (16) and TPHK (13) (Fig. 2e-g). Although dimethyl sulfide exhibited the highest OAV in all three samples (OAVs >100), its related “cooked vegetable-like” attribute showed relatively low sensory scores in TPHK and SCXLH. This might be attributed to perceptual interactions among odorants (Thomas-Danguin et al., 2014), particularly masking effects from co-existing floral compounds (Fukutani et al., 2023). While (*E,Z*)-2,6-nonadienal, typically associated with cucmber-like and green notes, was the second most potent odorant in LAGP (OAV = 153.1) and SCXLH (OAV = 76.2), (*E*)-*β*-ionone, known for its floral and violet-like aroma, ranked second in TPHK (OAV = 42.1). This difference might potentially contributing to the lower perceived intensity of green-like notes in TPHK.

Notably, 6-methyl-5-hepten-2-one—a compound with green and nutty attributes—exceeded its threshold only in LAGP (OAV = 13.9). Additionally, several green or malty-related odorants such as hexanal, (*Z*)-3-hexen-1-ol, and 2-methylbutanal exhibited markedly higher OAVs in LAGP, further reinforcing its green-like aroma profile. Compared with TPHK, SCXLH showed a broader presence of sweet-contributing compounds: *δ*-decalactone (OAV = 3.7) and *δ*-valerolactone (OAV = 1.9), a feature almost absent in TPHK (Fig. 1b, line 1).

Regarding floral odorants, the three samples shared several common compounds, yet varied in both the number and the relative contributions of these compounds based on OAVs. In LAGP, (*E,E*)-2,4-heptadienal was the predominant floral contributor (floral and fatty, OAV = 121.9). Geraniol, associated with a rose-like floral note, was dominant in SCXLH (OAV = 16.9), while (*E*)-*β*-ionone, known for its violet-like character, was most prominent in TPHK (OAV = 42.1). Despite the presence of shared floral compounds such as linalool, geraniol, (*E*)-*β*-ionone, jasmone, and epi-MeJA, their OAV values and relative importance differed considerably, further distinguishing the floral profiles of the teas. In particular, the OAV of the orchid-like epi-MeJA in TPHK and SCXLH was approximately nine- and seven-fold higher, respectively, than in LAGP, consistent with the sensory evaluation results. From a compositional perspective, both MeJA and epi-MeJA were present a substantially higher concentrations in TPHK and SCXLH compared to LAGP. However, the ratio between these two compounds didn't show a consistent trend among the samples (epi-MeJA/MeJA: 0.13 in LAGP, 0.12 in TPHK, and 0.09 in SCXLH), suggesting that their absolute concentrations, rather than their relative proportions, may play a more important role in shaping floral aroma profiles. Interestingly, although (*E*)-*β*-ionone was not the dominant odorant in LAGP, it ranked second with its OAV of 37.9, which was comparable to its value in TPHK (OAV = 42.1). This suggests what while (*E*)-*β*-ionone consistently contributes to the floral character across samples, its role in shaping the overall aroma profile of the floral green teas may be less pronounced compared to other key floral odorants.

**Fig. 3:**
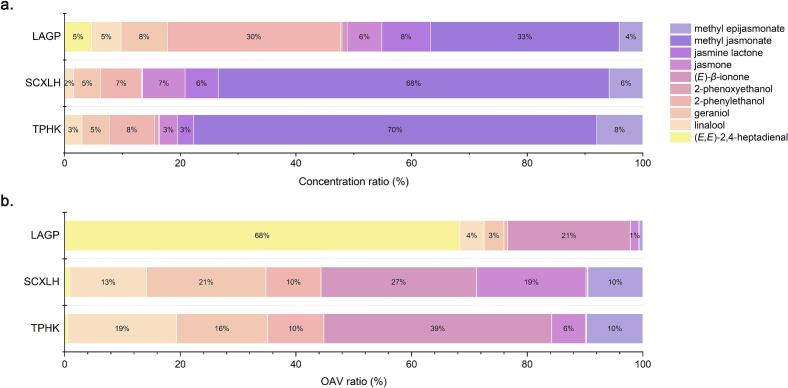
Comparison of concentration ratio (a) and OAV ratio (b) of floral-related odorants among three floral green teas. Proportions less than 1% are not shown in the figure. (For interpretation of the references to colour in this figure legend, the reader is referred to the web version of this article.)

When comparing the OAVs of floral-related odorants across the three green teas, distinct aroma-dominating patterns emergred (Fig. 3b). In LAGP, (*E,E*)-2,4-heptadienal overwhelmingly dominated the profile, contributing approximately 70% of the total floral OAVs, suggesting a more complex fatty and green nuance underlying its floral character. In contrast, (*E*)-*β*-ionone stood out in TPHK, accounting for 44% of its floral aroma contribution. Geraniol and linalool exhibited relatively balanced OAVs across all three samples, suggesting they may contribute to a common floral backbone in green teas. Interestingly, although epi-MeJA present a low concentrations, showed a non-neligible OAV contribution in SCXLH and TPHK, hinting at a possible role in nuanced floral expression. Overall, the OAV-based comparison reveals both shared contributors and sample-specific dominant odorants, emphasizing the complexity and diversity of floral perception among green teas.

### 3.4 Multivariate statistical analysis

#### 3.4.1 Partial least squares-discriminant analysis (PLS-DA)

**Fig. 4:**
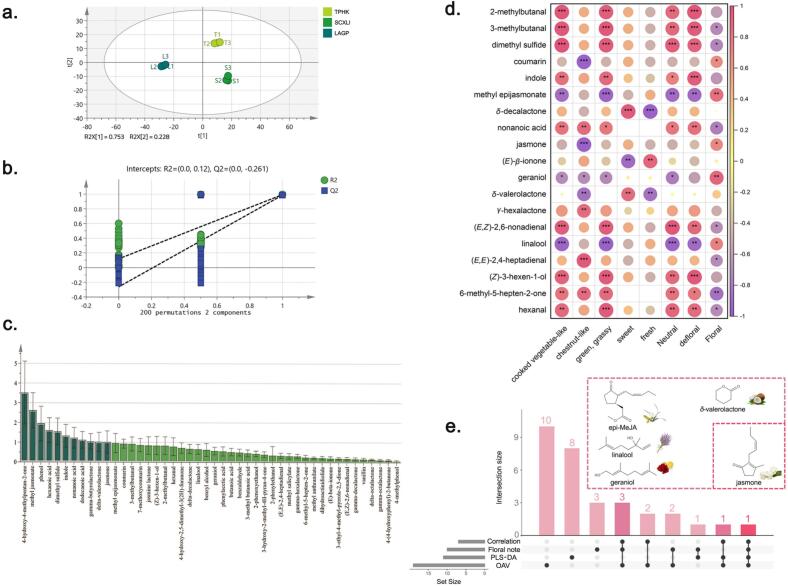
Multivariate analysis. a. PLS-DA score plot, b. permutation test (200 iterations) validation, and c. VIP scores of three floral green teas based on the concentrations of odorants, odorants with VIP > 1 were highlighted with dark green. d. Spearman correlation analysis between odor-active compounds (OAV > 1) and frequency-weighted sensory attributes (*, *p* < 0.05, **, *p* < 0.01, ***, *p* < 0.001). e. Upset plot of key odorants contribution to floral notes and differentiation across results from multiple criteria. (For interpretation of the references to colour in this figure legend, the reader is referred to the web version of this article.)

The PLS-DA model was built to achieve supervised discrimination (Fig. 4a-c). The model showed high goodness-of-fit and predictive ability, with R^2^X = 0.981, R^2^Y = 0.995, and Q^2^ = 0.991. The high Q^2^ value (0.991) indicates excellent predictive capacity of the model. Although the permutation test (Q^2^ intercept = −0.261) indicated a moderate overfitting risk, the negative intercept remained within an acceptable range, indicating the model retained statistical reliability.

The score plot (Fig. 4a) showed a clear separation among different samples. Based on the variable importance in projection (VIP) values, eleven odorants (VIP > 1, *p* < 0.05) were screened to be key compounds, including 4-hydroxy-4-methylpentan-2-one, MeJA, and phenol (Fig. 4d). Compared with the results of OAV, there were five odorants that have overlapping (i.e. dimethyl sulfide, indole, jasmone, nonanoic acid, and *δ*-valerolactone). Interestingly, epi-MeJA exhibited higher OAV but a VIP slightly below 1, which could be attribute to its much lower odor threshold compared with MeJA, showing the role of epi-MeJA with lower importance in differentiate samples but higher contribution to sample aroma profiles.

To achieve a more comprehensive understanding of the aroma differences among samples, both sensomics-guided techniques (sensory evaluation and GC–MS/O combined with AEDA) and statistical analyses (including OAV and multivariate statistical models) were employed. By comparing key odorants screened by FD, OAV, and PLS-DA (VIP > 1), five compounds—dimethyl sulfide, indole, jasmone, nonanoic acid, and *δ*-valerolactone—were found at the intersection of all three methods, suggesting their pivotal role in aroma differentiation.

Notably, nonanoic acid was only perceived in LAGP (FD = 16, moldy and fatty), while *δ*-valerolactone was unique to SCXLH (FD = 128, coconut-like and sweet), each with OAV > 1 in their respective samples, highlighting their contribution to characteristic aromas. In contrast, dimethyl sulfide, indole, and jasmone exhibited high FD factors in all three teas, but with significantly different OAV values, indicating their varying impacts across samples. Jasmone, in particular, was identified as a discriminating compound by PLS-DA (VIP > 1), reinforcing its role as both a character-impact compound and a potential marker of sample variation.

Further narrowing the analysis to floral-related odorants, 14 compounds (including one unidentified compound) were described as having “floral” or partly floral notes. Among them, (*E,E*)-2,4-heptadienal and nerol oxide exhibited high FD values only in LAGP, while dimethyl salicylate was uniquely prominent in SCXLH, and one unknown floral compound was found only in TPHK. Ten of these floral odorants were quantified, with (*E,E*)-2,4-heptadienal showing a remarkably high OAV in LAGP (OAV = 121.9). In addition, significant differences in the concentrations of linalool, jasmine lactone, methyl epijasmonate, and jasmone were observed among the three teas.

Taken together, these findings underscore the multifaceted importance of jasmone. It exhibited a high FD factor across all samples, was quantitatively distinct, and served as a variable of importance in PLS-DA. These characteristics suggest that jasmone not only contributes to the floral aroma profile but also plays a central role in differentiating green teas with distinct regional characteristics.

#### 3.4.2 Sensory weighting and correlation analysis

In complex food matrices, odor attributes rarely act in isolation but interact and complement each other in shaping the overall aroma profile. In addition, the three floral green teas exhibited differences in descriptors and their citation frequencies, which in turn influenced their sensory scores. To achieve a more precise comparison across samples and gain a relative balance between perceptual representativeness and method-logical rigor, sensory scores of odor attributes were recalculated by assigning weights based on their citation frequency among panelists, as the it reflects the importance of the attribute in perception to a certain extent. Although frequency-based weighting still present some subjectivity, this approach has been widely applied in lexicon development and descriptor prioritization (Drake & Civille, 2003; Suwonsichon, 2019). The weighted scoring system was then integrated into Spearman correlation analysis to further evaluate the relationships between sensory attributes and the odor-active compounds (OAV >1).

As shown in Fig. 4d, five odorants including coumarin (*r* = 0.667), epi-MeJA (*r* = 0.833), jasmone (*r* = 0.700), geraniol (*r* = 0.883), and linlool (*r* = 0.733) showed significant (*p* < 0.05) positive correlations with “floral, indicating their key roles in shaping the floral impression of the teas. Notably, linalool also showed a significant negative correlation (*r* = -0.883. *p* < 0.01) with “defloral” group. In line with previous studies, terpenoids were considered as key floral contributors in green tea and other plant-based matrices (Holt, Miks, de Carvalho, Foulquié-Moreno, & Thevelein, 2019; van Schie, Haring, & Schuurink, 2006; Yin et al., 2022).

While terpenoids such as linalool and geraniol served as the backbone of floral aroma in green teas, subtle differences among the teas were further shaped by odorants with more specific floral notes. For instance, epi-MeJA has been reported as a characteristic odorant for orchid-like aroma (Feng et al., 2020), showed higher concentration in TPHK and SCXLH, consistent with the descriptor frequency results, and its higher OAV in TPHK reinforced the orchid-like intensity.

Besides the odorants that have obvious floral related odor quality description, some lactones which showed moderate positive correlations with sweet odor notes, implying a complementary effect that might indirectly support floral expression (*δ*-valerolactione: *r* = 0.317, *p* = 0.41, and coumarin: *r* = 0.667, *p* < 0.05). For example, SCXLH, with higher concentration of lactones, get higher citation of descriptors like “coconut-like” and “milk-like”. In contrast, aldehydes such as 2-methylbutanal, 3-methylbutanal, and hexanal correlated positively with green/grassy which was classified as de-floral note, suggesting a potential masking or suppressing effect on floral perception.

These molecular-sensory associations translated into distinct sensory profiles among the three green teas: TPHK and SCXLH, enriched in terpenoids and lactones, were characterized by stronger and more specific floral notes, while LAGP showed a relatively weaker floral intensity, likely due to the presence of aldehydes in higher concentrations.

Overall, the floral differentiation among the three teas was not solely driven by the floral-positive odorants, but also by the dynamic balance between enhancing odorants (lactones) and antagonistic odorants (aldehydes, sulfur compounds), reflecting the complexity of aroma modulation in green tea, where the final sensory impression emerges from both reinforcement and suppression effects within the aroma composition.

As shown in Fig. 4e, five odorants were identified through the integration of four complementary selection criteria: descriptive odor quality (“floral note”), OAV (> 1), PLS-DA (VIP > 1), and significant positive correlation with the “floral” or other floral-related attributes, including epi-MeJA, linalool, geraniol, *δ*-valerolactone, and jasmone. These results indicated that the floral expression in the three Anhui green teas is not driven by a single compound but by a group of high-impact odorants that are both abundant (high OAV) and discriminative (high VIP and positive correlation).

Linalool, geraniol, and epi-MeJA appeared in three analytical dimensions (except PLS-DA), further supporting their role as backbone floral odoratns shared across the three teas. *δ*-Valerolactone, in contrast, was present in three dimentsions but not the “floral note” category, suggesting that it contributed indirectly by modulating floral style through potential synergistic interactions between “sweet” and “floral” attributes. Notably, jasmone was the only odorant present in all four analytical dimensions, indicating its important role in both contributing to the floral impression and differentiating floral types depending on its concentration. The compounds highlighted by the UpSet plot provide focused targets for future validation (e.g., recombination or omission tests and enantiomeric analysis) to confirm their authentic roles in shaping different floral styles in green tea.

### 3.5 Potential factors underlying floral aroma differentiation

The distinct floral styles of the three Anhui green teas can be attributed to the combined influence of cultivar-specific metabolic traits, terroir-driven precursor accumulation, and regionally characteristic processing techniques, which collectively shape the final odorant profiles at multiple levels.

From a compositional perspective, cultivar serves as the fundamental determinant of aroma precursor composition. The abundance and type of precursors are closely associated with genetic background (Zheng, Li, Xiang, & Liang, 2016), thereby defining the upper limit for subsequent aroma formation. This precursor pool is further modulated by environmental factors, including climate and soil conditions (Huang et al., 2024; Li et al., 2024). In addition, cultivar-dependent differences in gene expression related to key enzymes may influence aroma formation pathways; for example, the expression of *CsJMT* has been reported to promote MeJA formation via the linolenic acid pathway (Feng et al., 2021). Leaf maturity also plays a role in determining the potential for aroma development (Zhang et al., 2026).

From a processing perspective, both enzymatic reactions during the post-harvest stage and thermal reactions during fixation and drying contribute to the formation and transformation of odorants (Ho, Zheng, & Li, 2015). Notably, Anhui green teas, including the samples analyzed in this study, typically undergo multi-stage baking, which may provide a relatively controlled thermal environment that facilitates continuous transformation and accumulation of aroma compounds (Feng et al., 2023; Yu et al., 2024; Zhang et al., 2024).

Beyond the contribution of individual compounds, the overall floral perception is further shaped by interactions among odorants. Synergistic effects between floral and sweet-related compounds (e.g., lactones) may enhance floral expression (Xiao et al., 2024), while green or sulfur-containing compounds may exert masking effects, suppressing certain aroma notes (Fukutani et al., 2023).

Overall, the differentiation of floral aroma among TPHK, SCXLH, and LAGP reflects a dynamic balance shaped by precursor composition, environmental conditions, and processing practices, ultimately leading to distinct sensory profiles.

## 4 Conclusion

In this study, the aroma profiles of three representative floral-type Anhui green teas were systematically compared by integrating sensory evaluation with sensomics-guided analysis and multivariate statistical analysis. A total of 59 odorants were identified and quantitated, among which linalool, geraniol, epi-MeJA, *δ*-valerolactone, and jasmone were consistently identified as key contributors to floral differentiation. Notably, jasmone was the only odorant supported across all analytical dimensions, indicating its role in both contributing to the floral note and intensity, and distinguishing the floral types. Moreover, correlation analysis between odor-active compounds (OAV > 1) and weighted-scored sensory attributes revealed that floral perception was not solely enhanced by positive floral volatiles, but also modulated by sweet note which, in this case, contributed by *δ*-valerolactone.

These findings elucidated the molecular basis for the distinct floral characteristics of the investigated Anhui green teas. The aroma diversity among them can be attributed to a model: a shared floral backbone primarily contributed by odorants like linalool and geraniol, that modulate florla intensity, and signature markers such as jasmone, which shape specific floral styles. This study also demonstrated the value of combining sensory-guided analysis with multivariate statistics, providing a chemical perspective moving from subjective description to a more objective and chemically grounded framework for understanding floral aroma in green tea. Nevertheless, although key odorants were identified based on multiple analytical criteria, further confirmation by recombination and omission experiments would provide more direct validation of their sensory contributions and will be considered in future studies. Overall, these findings provided practical chemical insights for the targeted processing of Anhui green teas, enabling to optimize and standardize their desirable floral attributes.

## Abbreviations

**Table:** 

GC–MS/O	gas chromatography-olfactometry/mass spectrometry
TPHK	*Taiping Houkui*
SCXLH	*Shucheng Xiaolanhua*
LAGP	*Lu’an Guapian*
SAFE	solvent-assisted flavor evaporation
HS-SPME	headspace solid-phase microextraction
AEDA	aroma extract dilution analysis
ODT	odor detection threshold
OAV	odor activity value
PLS-DA	partial least squares discriminant analysis
DCM	dichloromethane
DEE	diethyl ether
nerol oxide	3,6-Dihydro-4-methyl-2-(2-methyl-1-propenyl)-2H-pyran
MeJA	methyl jasmonate
epi-MeJA	methyl epijasmonate
FID	flame ionization detector
trans-linalool oxide (IV)	(3*R*,6*S*)-6-ethenyl-2,2,6-trimethyloxan-3-ol
ODP	olfactory detection port
FD	flavor dilution
MS-EI	mass spectrometer electron impact mode
EWM	entropy weight method
TF-IDF	term frequency-inverse document frequency
SD	standard deviation
ANOVA	analysis of variance
EIC	extracted ion chromatograms
PC	principal component
VIP	variable importance in projection

## CRediT authorship contribution statement

**Jieyao Yu:** Writing – original draft, Visualization, Validation, Methodology, Investigation, Formal analysis, Data curation, Conceptualization. **Tianyuan Yang:** Writing – review & editing, Validation, Formal analysis, Data curation. **Qian Chen:** Validation, Methodology, Investigation. **Gang Wang:** Validation, Methodology, Investigation. **Dongzhu Huang:** Visualization, Validation, Methodology, Investigation. **Małgorzata Starowicz:** Writing – review & editing, Supervision. **Xiaochun Wan:** Supervision, Funding acquisition, Conceptualization. **Xiaoting Zhai:** Writing – review & editing, Visualization, Validation, Supervision, Investigation, Funding acquisition, Conceptualization.

## Ethical statement

The authors affirm that the work was conducted in compliance with The Code of Ethics of the World Medical Association (Declaration of Helsinki) for experiments involving human. Ethical approval for sensory evaluation is not mandated by national regulations, and no formal human ethics committee or documentation process is required. The study adhered to protocols ensuring participant rights and privacy were safeguarded, including voluntary participation, transparent communication of study risks, verbal consent, non-disclosure of participant data without consent, and the freedom to withdraw from the study at any time.

## Declaration of competing interest

The authors declare that they have no known competing financial interests or personal relationships that could have appeared to influence the work reported in this paper.

## Data Availability

Data will be made available on request.
