# Supplementary material for: Sensory-guided and statistical characterization of key odorants driving floral differentiation in three Anhui green teas
Source: Food Chem X. 2026 Apr 30;36:103936. doi: 10.1016/j.fochx.2026.103936 (PMC13153592; doi:10.1016/j.fochx.2026.103936)
Supplement: Supplementary material [file mmc1.docx]

**Table S1**. Detailed information of the tea samples

| **No.** | **Product name^a^** | **Sample grade^b^** | **Production date** | **Place of origin** | **Purchase source** |
| --- | --- | --- | --- | --- | --- |
| 1 | TPHK-1 | Premium (Ji pin) | April, 2024 | Houkeng, Huangshan, Anhui, China | Local tea producer (Houkeng) |
| 2 | TPHK-2 | Premium (Ji pin) | April, 2024 | Houkeng, Huangshan, Anhui, China | Liubaili Houkui Tea Co., Ltd. |
| 3 | TPHK-3 | Super grade (Te ji) | April, 2023 | Huangshan, Anhui, China | Xie Yuda Tea Co., Ltd. |
| 4 | LAGP-1 | Premium (Jing pin) | April, 2024 | Yu’an Distric, Lu’an, Anhui, China | Huiliu (Anhui Lu’an Guapian Tea Industry Co., Ltd.) |
| 5 | LAGP-2 | Super grade 1 (Te yi) | April, 2024 | Yu’an Distric, Lu’an, Anhui, China | Huiliu (Anhui Lu’an Guapian Tea Industry Co., Ltd.) |
| 6 | SCXLH-1 | Super grade 1 (Te yi) | April, 2024 | Shucheng, Lu’an, Anhui, China | Jiuyiliu Tea Co., Ltd. |
| 7 | SCXLH-2 | Super grade 1 (Te yi) | Spring 2024 | Shucheng, Lu’an, Anhui, China | Local tea producer (Shucheng) |

^a^Several commercial samples were collected for each tea type. Based on preliminary sensory evaluation, one representative sample from each category (TPHK-1, LAGP-1, and SCXLH-1) was selected and used for the subsequent analyses.

^b^Sample grades were based on the labels provided by the manufacturers and further evaluated by sensory assessment with reference to relevant Chinese national (GB) and regional (DB) standards.

**Table S2**. Standard curves for quantitation.

| **No.^a^** | **Compounds** | **Selected ions (*m/z*)^b^** | **Standard curve** | **R^2^** |
| --- | --- | --- | --- | --- |
| ethyl caprate as internal standard | | | | |
| 1 | hexanal | 44 | y = 7.5745x + 0.0982 | 1 |
| 5 | 6-methyl-5-hepten-2-one | 43 | y = 2.6581x + 0.0775 | 0.9998 |
| 7 | 4-hydroxy-4-methylpentan-2-one | 43 | y = 2.767x - 0.1197 | 0.9999 |
| 8 | (*Z*)-3-hexen-1-ol | 41 | y = 3.295x + 0.0156 | 0.9999 |
| 12 | (*E,E*)-2,4-heptadienal | 81 | y = 1.4093x - 0.0538 | 0.9995 |
| 13 | benzaldehyde | 77 | y = 1.4641x - 0.0193 | 0.9998 |
| 14 | linalool | 71 | y = 2.4084x + 0.1052 | 0.9998 |
| 16 | (*E,Z*)-2,6-nonadienal | 41 | y = 2.7497x - 0.2082 | 0.9998 |
| 18 | butanoic acid | 60 | y = 5.4031x - 0.9222 | 0.9995 |
| 19 | *γ*-butyrolactone | 42 | y = 0.1162x + 0.032 | 1 |
| 20 | 3-methyl butanoic acid | 60 | y = 1.889x - 0.0577 | 1 |
| 21 | *γ*-hexalactone | 85 | y = 1.1505x + 0.0206 | 1 |
| 23 | methyl salicylate | 120 | y = 0.9228x - 0.0259 | 0.9994 |
| 24 | *δ*-valerolactone | 42 | y = 3.805x + 0.1466 | 0.9999 |
| 25 | hexanoic acid | 60 | y = 2.1421x - 0.1765 | 0.9994 |
| 26 | geraniol | 69 | y = 1.334x - 0.0562 | 0.9995 |
| 27 | benzyl alcohol | 79 | y = 2.0373x - 0.1034 | 0.9961 |
| 28 | 2-phenylethanol | 91 | y = 0.7512x - 0.0174 | 0.9996 |
| 29 | *γ*-octalactone | 85 | y = 0.6242x + 0.0389 | 0.9998 |
| 30 | (*E*)-*β*-ionone | 177 | y = 1.065x - 0.0204 | 0.9998 |
| 31 | jasmone | 164 | y = 3.4683x - 0.1224 | 0.9998 |
| 32 | *δ*-octalactone | 99 | y = 0.6009x - 0.0098 | 0.9959 |
| 33 | 3-hydroxy-2-methyl-4*H*-pyran-4-one | 126 | y = 1.8782x - 0.0415 | 0.9998 |
| 34 | phenol | 94 | y = 0.8384x + 0.0078 | 1 |
| 34 | 4-hydroxy-2,5-dimethyl-3(2*H*)-furanone | 43 | y = 7.7364x - 0.4235 | 1 |
| 38 | 4-methylphenol | 107 | y = 1.1045x - 0.0014 | 1 |
| 39 | 2-phenoxyethanol | 94 | y = 0.9011x + 0.0277 | 0.9995 |
| 41 | *γ*-decalactone | 85 | y = 1.114x + 0.0704 | 0.9989 |
| 43 | nonanoic acid | 60 | y = 5.8266x - 0.7001 | 0.9998 |
| 44 | *δ*-decalactone | 99 | y = 4.7285x - 0.9113 | 0.9968 |
| 45 | methyl anthranilate | 119 | y = 1.1259x + 0.0609 | 0.9997 |
| 46 | jasmine lactone | 99 | y = 1.2143x - 0.0543 | 0.9996 |
| 48 | 3-ethyl-4-methyl-pyrrole-2,5-dione | 139 | y = 1.2509x + 0.0869 | 0.9997 |
| 50 | methyl jasmonate | 83 | y = 3.3443x - 0.0918 | 0.9998 |
| 51 | dihydroactinidiolide | 111 | y = 1.4393x + 0.0191 | 1 |
| 52 | methyl epijasmonate | 83 | y = 117.24x - 2.5084 | 0.9999 |
| 53 | indole | 117 | y = 0.468x + 0.0088 | 1 |
| 54 | coumarin | 146 | y = 1.1389x + 0.125 | 0.9962 |
| 55 | dodecanoic acid | 73 | y = 88.477x - 29.223 | 0.9963 |
| 56 | phenylacetic acid | 91 | y = 1.3513x - 0.0825 | 0.9999 |
| 57 | vanillin | 151 | y = 0.9842x - 0.0288 | 1 |
| 58 | 7-methoxycoumarin | 176 | y = 1.2207x + 0.038 | 0.9998 |
| 59 | 4-(4-hydroxyphenyl)-2-butanone | 107 | y = 1.1983x - 0.0078 | 0.9998 |
| stable isotopes as internal standard | | | | |
| HS-2 | dimethyl sulfide | 63 | y = 0.9518x - 0.0424 | 0.9998 |
|  | [^2^*H*_6_]dimethyl sulfide | 69 |  |  |
| HS-4 | 3-methylbutanal | 87 | y=1.0353x+0.1356 | 0.9999 |
|  | [^2^*H*_2_]3-methylbutanal | 89 |  |  |
| HS-5 | 2-methylbutanal | 87 | y=1.0183x-0.0145 | 0.9968 |
|  | [^2^*H*_2_]3-methylbutanal | 89 |  |  |

^a^The numbers are correspond to Table 1, with sorting based on retention index.

^b^Ions monitored for quantitation

**Table S3.** The scores, citation frequency and grouping of attributes of three green tea samples.

| **Group** | **Floral-related** | | | | | | | | | | **Defloral-related** | | | | | | | | **Neutral** | | | |
| --- | --- | --- | --- | --- | --- | --- | --- | --- | --- | --- | --- | --- | --- | --- | --- | --- | --- | --- | --- | --- | --- | --- |
| Attribute^a^ | orchid-like | | tender | | fresh | | floral | | sweet | | hay-like | | cooked vegetable-like | | green, grassy | | fatty | | chestnut-like | | seaweed-like | |
| Sample | score | f^b^ | score | f | score | f | score | f | score | f | score | f | score | f | score | f | score | f | score | f | score | f |
| TPHK | 2.83 | 15 | N/A^c^ | 5 | 1.66 | 13 | N/A | 0 | 0.95 | 11 | 0.92 | 8 | 1.21 | 7 | 1.07 | 15 | N/A | 2 | 0.87 | 12 | N/A | 1 |
| SCXLH | 2.36 | 14 | 2.07 | 7 | N/A | 10 | N/A | 0 | 1.23 | 8 | N/A | 4 | 1.74 | 8 | 1.35 | 15 | N/A | 5 | 0.68 | 8 | 1.27 | 5 |
| LAGP | N/A | 1 | N/A | 2 | 1.11 | 5 | 2.18 | 7 | 1.17 | 9 | N/A | 6 | 2.16 | 11 | 1.68 | 15 | 1.15 | 9 | 1.54 | 9 | N/A | 3 |

^a^Average sensory evaluation score provided by 15 panelists.

^b^f represents the citation frequency of the descriptor.

^c^N/A indicates that the attribute was not scored by the panelists due to its low citation frequency.

**Table S4**. The aroma descriptions from traditional sensory evaluation.

| **Sample** | **Aroma description** |
| --- | --- |
| TPHK | Pronounced intense orchid-like aroma, fresh and rich scent profile with a slight roasted chestnut note. |
| SCXLH | Bright and persistent orchid-like aroma with a faint tender chestnut note and a noticeable fresh-green note. |
| LAGP | Delicate floral aroma with dominant roasted chestnut and pronounced toasty aroma. |

**
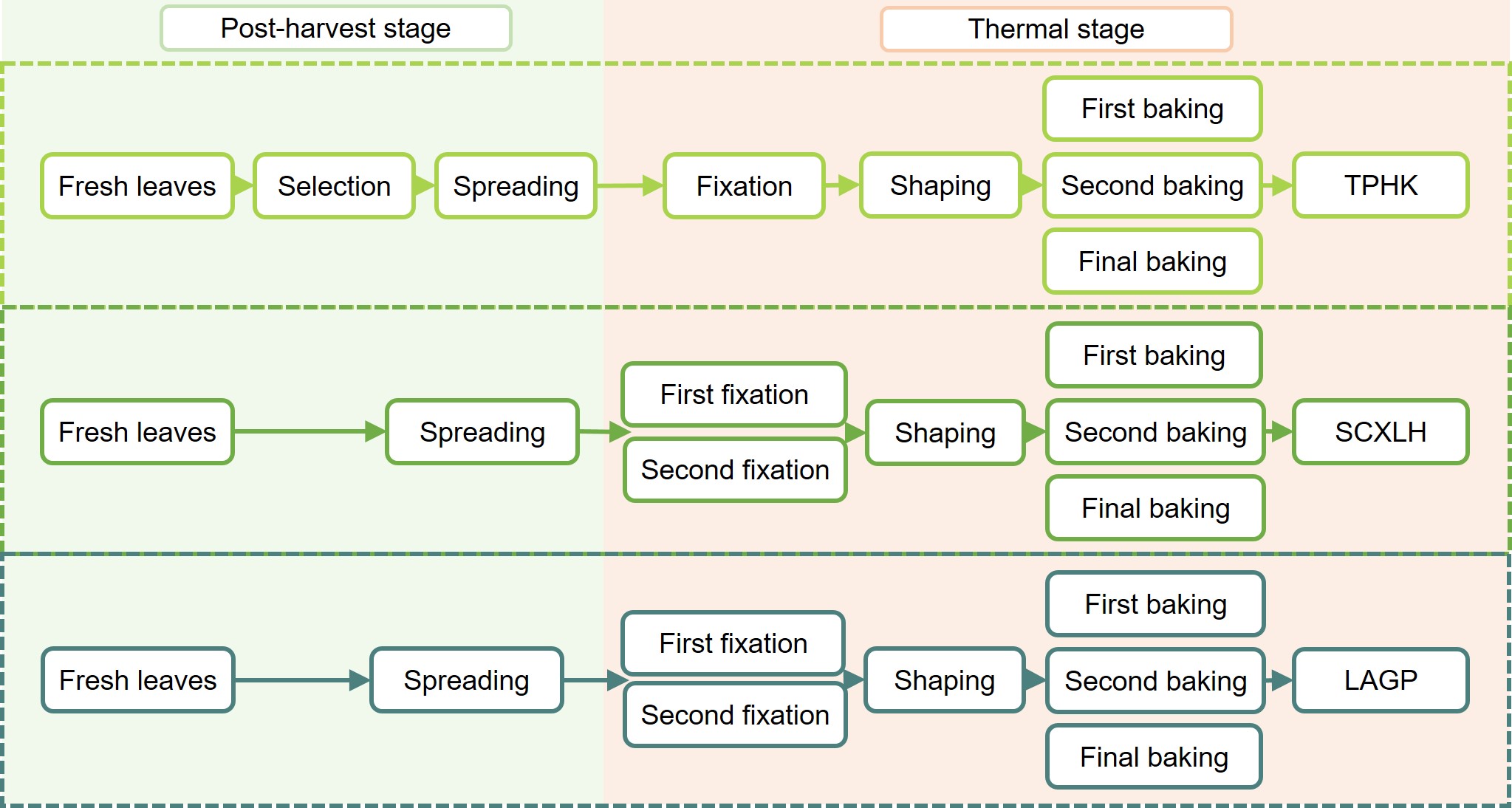
Figure S1.** Schematic flowchart of the processing procedures for the three characteristic Anhui green teas. TPHK: *Taiping Houkui*, SCXLH: *Shucheng Xiaolanhua*, LAGP: *Lu’an Guapian*.
